# Comparison between micro- and nanosized copper oxide and water soluble copper chloride: interrelationship between intracellular copper concentrations, oxidative stress and DNA damage response in human lung cells

**DOI:** 10.1186/s12989-017-0209-1

**Published:** 2017-08-01

**Authors:** Bettina Maria Strauch, Rebecca Katharina Niemand, Nicola Lisa Winkelbeiner, Andrea Hartwig

**Affiliations:** Department of Food Chemistry and Toxicology, Karlsruhe Institute of Technology (KIT), Institute for Applied Biosciences, Adenauerring 20a, 76131 Karlsruhe, Germany

**Keywords:** Copper oxide nanoparticles, Oxidative stress, Gene expression profiling, High-throughput RT-qPCR, DNA damage response, Copper uptake and intracellular distribution

## Abstract

**Background:**

Nano- and microscale copper oxide particles (CuO NP, CuO MP) are applied for manifold purposes, enhancing exposure and thus the potential risk of adverse health effects. Based on the pronounced in vitro cytotoxicity of CuO NP, systematic investigations on the mode of action are required. Therefore, the impact of CuO NP, CuO MP and CuCl_2_ on the DNA damage response on transcriptional level was investigated by quantitative gene expression profiling via high-throughput RT-qPCR. Cytotoxicity, copper uptake and the impact on the oxidative stress response, cell cycle regulation and apoptosis were further analysed on the functional level.

**Results:**

Cytotoxicity of CuO NP was more pronounced when compared to CuO MP and CuCl_2_ in human bronchial epithelial BEAS-2B cells. Uptake studies revealed an intracellular copper overload in the soluble fractions of both cytoplasm and nucleus, reaching up to millimolar concentrations in case of CuO NP and considerably lower levels in case of CuO MP and CuCl_2_. Moreover, CuCl_2_ caused copper accumulation in the nucleus only at cytotoxic concentrations. Gene expression analysis in BEAS-2B and A549 cells revealed a strong induction of uptake-related metallothionein genes, oxidative stress-sensitive and pro-inflammatory genes, anti-oxidative defense-associated genes as well as those coding for the cell cycle inhibitor p21 and the pro-apoptotic Noxa and DR5. While DNA damage inducible genes were activated, genes coding for distinct DNA repair factors were down-regulated. Modulation of gene expression was most pronounced in case of CuO NP as compared to CuO MP and CuCl_2_ and more distinct in BEAS-2B cells. GSH depletion and activation of Nrf2 in HeLa S3 cells confirmed oxidative stress induction, mainly restricted to CuO NP. Also, cell cycle arrest and apoptosis induction were most distinct for CuO NP.

**Conclusions:**

The high cytotoxicity and marked impact on gene expression by CuO NP can be ascribed to the strong intracellular copper ion release, with subsequent copper accumulation in the cytoplasm and the nucleus. Modulation of gene expression by CuO NP appeared to be primarily oxidative stress-related and was more pronounced in redox-sensitive BEAS-2B cells. Regarding CuCl_2_, relevant modulations of gene expression were restricted to cytotoxic concentrations provoking impaired copper homoeostasis.

**Electronic supplementary material:**

The online version of this article (doi:10.1186/s12989-017-0209-1) contains supplementary material, which is available to authorized users.

## Background

Due to desired material properties such as high temperature superconductivity, artificially synthesized CuO nanoparticles (NP) are increasingly applied in industry, for example as catalysts, additives in plastics and metal coatings, but also as antimicrobial agents (summarized in [1]). CuO microparticles (MP) are precursors during pigment production [2]. Furthermore, CuO fumes including ultrafine particles are generated in metal refining and processing [3–5].

In biological systems, copper represents the third common trace element next to zinc and iron. It serves as catalytic cofactor in more than 30 metalloenzymes like superoxide dismutase (SOD), cytochrome C oxidase (COX), lysyl oxidase, dopamine-β-hydroxylase and ceruloplasmin, thus playing critical roles within manifold biochemical pathways [6]. While the redox-activity is required for essential functions, copper ions may also be toxic due to the catalysis of Fenton-type reactions, which may lead to the formation of highly reactive hydroxyl radicals, provoking oxidative damage to lipids, proteins and DNA [7]. To ensure essential functions and to largely prevent toxic reactions, both systemic as well as cellular copper homoeostasis are tightly regulated by copper binding to proteins and/or to small chelating molecules, preventing the existence of “free” copper ions. On the systemic level, copper homeostasis is regulated via absorption in the small intestine and via storage, distribution and biliary excretion by the liver, avoiding overload or deficiency. Similarly, within cells, copper levels and protein binding are strictly controlled via uptake, storage, transfer and export proteins. Genetically determined disturbances in copper homeostasis lead to severe disease syndromes like Wilson’s or Menke’s disease [8]. Nevertheless, copper overload can also occur due to excessive uptake, for example via dietary supplements, or due to non-physiological exposure routes. While copper uptake occurs usually via food and drinking water by the oral route, which is tightly regulated, the situation may be different in case of inhalation of copper fumes or aerosols in exposed workers, leading potentially to cellular and/or systemic copper overload.

Different working groups reported an enhanced cytotoxic and genotoxic potential as well as a strong pro-inflammatory response of CuO NP in various, mainly lung-associated cell culture models when compared to other metal-based nanoparticles [9–11] as well as to bigger particles or water soluble copper compounds [12–15]. Thus, besides the elemental composition different physico-chemical characteristics appear to contribute to the pronounced toxicity. Important features may include size, morphology, solubility, surface area as well as the ability of the particles to associate with biomolecules from the environment to form a protein corona [16], with potential impact on interactions of the particles with cellular membranes, uptake and intracellular bioavailability, finally leading to elevated levels of ROS.

One explanation originally proposed by Limbach and co-authors in 2007 in case of Co_3_O_4_ nanoparticles and since then strengthened in several studies e.g. by Karlsson and co-workers for CuO NP has been termed the “Trojan horse type mechanism”. It proposes the endocytotic uptake of the particles and the processing of the resulting vesicles into acidic lysosomes, thus affecting the degree of metal release from the particles and provoking potential intracellular overload conditions [12–15, 17–19]. Supporting this theory, many NP have been reported to use endocytotic mechanisms as major import pathway into the cell [20–23], including nanosized CuO in A549 cells [19].

However, many aspects still need to be elucidated. Thus, while the high cytotoxicity of CuO NP has been repeatedly demonstrated, less attention has been given to the impact on genomic stability. Furthermore, also the degree and intracellular distribution of deliberated copper ions is still not known. With respect to the latter, copper uptake has frequently been determined in whole cells, baring the risk of irrelevant results due to remaining and barely removable particles at the outer membrane.

Within the present study, we used adenocarcinoma A549 and BEAS-2B lung cells since the lung is the major target organ of CuO NP toxicity upon inhalative exposure to assess the impact on the DNA damage response system and to link it to intracellular copper levels upon copper particle or water soluble copper exposure. In a previous study of our working group, a detailed investigation on the cyto- and genotoxicity as well as copper uptake of CuO NP as compared to CuO MP and CuCl_2_ was performed in A549 cells, demonstrating pronounced cytotoxicity in case of CuO NP and CuCl_2_, but no effect in case of CuO MP. Furthermore, apoptosis induction as well as the induction of DNA strand breaks analysed in the model cell line HeLa S3 were restricted to CuO NP. Also, strongest impairment of the DNA damage signalling reaction poly(ADP-ribosylation) mediated predominatly by poly(ADP-ribose) polymerase 1 was observed in case of CuO NP. Finally, highest intracellular copper levels, both in the cytoplasm and the nucleus, were found in case of CuO NP [14]. Following up on this investigation in A549 cells, the central aspect of the present study was to elucidate the impact of all three copper compounds on gene expression profiles by using a recently established high-throughput RT-qPCR system via the BioMark™ device. This procedure enables the parallel assessment of the impact of 96 samples on 95 genes crucial for maintaining genomic stability, consisting of selected genes coding for stress response, DNA damage response, specific DNA repair factors, cell cycle control, apoptosis and mitotic signalling [24]. The effects of CuO NP were compared with those evoked by CuO MP and water soluble CuCl_2_ in identical mass doses to differentiate between particle, nano-particle and copper-mediated effects. In addition to A549 cells we included BEAS-2B cells as a second lung cell model. Both cell lines display advantages and disadvantages; A549 cells are p53 proficient but as a tumor cell line they have lost some characteristics of the original epithelial type 2 cells and they display some persistent basal signalling deregulation. In contrast, BEAS-2B cells are non-tumorigenic, but functionally p53 deficient due to the immortalization [25]. Thus, the comparison of the two cell lines with different transformation status and the knowledge about their respective limitations may provide valuable hints for evaluating and interpreting the effects on gene expression profiles. Finally, different outcomes on the transcriptional response were followed up on the functional level, such as the impact on the oxidative stress response, cell cycle regulation and apoptosis.

## Results

### Particle characterization

The particulate copper compounds CuO NP and CuO MP were characterized in detail with respect to size, surface area and charge, chemical composition, crystallinity and morphology via dynamic light scattering (DLS), scanning electron microscopy (SEM), Brunauer-Emmet-Teller (BET) analysis, zeta potential (ZP), inductively coupled plasma mass spectrometry (ICP-MS), energy-dispersive X-ray spectroscopy (EDX) as well as X-ray diffraction (XRD) as previously described. Briefly, CuO NP as well as CuO MP were of high purity and of the same composition, free of endotoxins and did not alter the pH of cell culture media. CuO NP were approximately spherical, displayed a narrow size range (20–200 nm), showed a surface area of 17.23 m^2^/g with a calculated average diameter of 55 nm and were agglomerated in fluids. DLS measurements revealed a size distribution around 146 nm and a ZP of −13.1 mV in DMEM/FCS. CuO MP had a size distribution of 500 nm −10 μm, a surface area of 0.74 m^2^/g and a calculated average of 1289 nm. DLS analysis was not possible due to fast sedimentation of the particles [14].

### Cytotoxicity

**Fig. 1:**
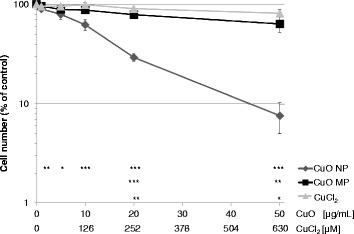
Cytotoxicity of CuO NP, CuO MP and CuCl_2_ **.** Cytotoxicity was determined via cell number after 24 h treatment with the different copper compounds in BEAS-2B cells. Shown are mean values of six determinations derived from three independent experiments ± SD. Statistically significant different from control: *p ≤ 0.05*,* **p ≤ 0.01*,* ***p ≤ 0.001 (ANOVA-Dunnett’s T test)

The impact on the cell number of BEAS-2B cells after 24 h incubation with CuO NP, CuO MP and CuCl_2_ was determined as a measure of cytotoxicity and to establish appropriate incubation conditions for subsequent analyses (Fig. 1). This approach was chosen since these cells do not form colonies and an interference by copper ions and copper-based particles with dye-based toxicity assays has been reported previously [26]. CuO NP provoked a strong concentration-dependent decrease of cell number. Thus, in case of 5 μg/mL a reduction to 79% compared to control was observed, reaching 30% and 10% in case of 20 μg/mL or 50 μg/mL, respectively. In contrast, incubation with CuO MP resulted in cell numbers above 80% up to 20 μg/mL and only in case of 50 μg/mL a pronounced reduction to 64% was observed. CuCl_2_ was only weakly cytotoxic with a maximum decrease of cell number to 81% throughout the analysed concentration range. In A549 cells the cytotoxicity of the same compounds has been investigated by colony forming ability and published previously; here, most pronounced cytotoxicity was also observed in case of CuO NP followed by CuCl_2_, whereas CuO MP were not cytotoxic throughout the analysed concentration range. As opposed to BEAS-2B cells, CuCl_2_ exhibited starting cytotoxicity at the lowest concentration tested, i.e. 63 μM corresponding to 5 μg/mL [14]. On the basis of these cytotoxicity results, subsequent investigations were performed mainly up to 20 μg/mL CuO and 252 μM CuCl_2_, respectively.

### Cellular uptake and intracellular distribution of copper

Cellular uptake and intracellular distribution of copper was analysed after 24 h incubation with different concentrations of CuO NP, CuO MP and CuCl_2_ via graphite furnace atomic absorption spectrometry (GF-AAS). When working with particles, one problem consists in the fact that particles attach and stick to the outer membrane, which may provide artefacts when determining the intracellular copper content. Thus, a special procedure was applied to quantify the copper content in the soluble fractions of the whole cell, the cytoplasm and the nucleus [14, 27].

**Fig. 2:**
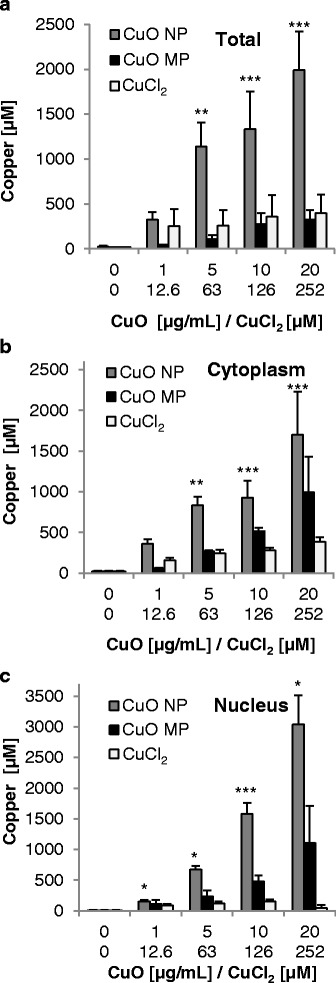
Cellular copper uptake and intracellular copper distribution after treatment with CuO NP, CuO MP and CuCl_2_. Copper content was determined in the soluble fraction of the total cell **(a)**, the cytoplasm **(b)** and the nucleus **(c)** in BEAS-2B cells after 24 h treatment with the different copper compounds via GF-AAS. Shown are mean values of three independent experiments + SD (**(a)** and CuO NP (**b,c**)), mean values of six independent experiments + SD (10 μg/mL CuO NP (**b,c)**) or mean values of two independent experiments + R/2 **(**CuO MP and CuCl_2_ (**b, c**)**)**. Statistically significant different from control: *p ≤ 0.05*,* **p ≤ 0.01*,* ***p ≤ 0.001 performed in case of (**a**) and CuO NP (**b**, **c**) (ANOVA-Dunnett’s T test). 20 μg/mL CuO are equal to 252 μM Cu^2+^. n.d.: not determined

As shown in Fig. 2a, the basal copper concentration in BEAS-2B cells was found to be 20 μM. Treatment with CuO NP resulted in a pronounced dose-dependent accumulation of intracellular copper. As low as 1 μg/mL provoked an 18-fold accumulation to 330 μM copper. After treatment with 5 μg/mL and higher intracellular concentrations up to 2 mM were observed. CuO MP also provoked a concentration-dependent but less pronounced increase in intracellular copper, resulting in a maximum of 330 μM. Incubation with CuCl_2_ was found to result in almost constant intracellular levels of 250–400 μM copper within the applied concentration range up to 252 μM.

The analysis of the cytoplasmic copper levels revealed a similar picture. Treatment with CuO NP resulted in copper levels of up to 1.7 mM in case of 20 μg/mL. Treatment with CuO MP displayed less pronounced dose-dependent increases in cytoplasmic copper as compared to CuO NP; at the highest dose, however, copper levels were higher than in the whole cells. This may be due to higher variations due to inhomogeneous particle uptake; thus, small differences in endocytosed particle numbers would be expected to result in pronounced differences in intracellular copper levels. Treatment with CuCl_2_ again revealed comparatively small increases up to 400 μM (Figure 2b). The basal copper concentration within the nuclear fraction of BEAS-2B cells was about 11 μM. Most strikingly, almost no increase in copper concentrations was observed in case of CuCl_2_, while CuO NP led to a markedly increased accumulation of nuclear copper up to 3 mM; this was even higher when compared to the cytoplasm. Intermediate levels were found in case of CuO MP. The cytoplasmic and nuclear copper levels add very well to the copper accumulation of the total cell. Since the volume of the nucleus accounts for only 10% of the cell volume, the cytoplasmic concentration should be close to the total cell volume. For example, in case of 20 μg/mL CuO NP respective values were 2000 μM (total), 1700 μM (cytoplasm), 3000 μM (nucleus); multiplied by the relative volumes results in an average concentration of 1830 μM, which fits quite reasonable to the copper content of the total cell. Similar agreements were found for the other concentrations applied.

Additionally, we quantified the copper content in whole cells including the outer membrane to get an impression of particles sticking to it, applying doses of 10 μg/mL CuO NP and CuO MP (Additional file 1). When not excluding the membrane, copper concentration was elevated more than 3.5-fold from 1350 μM up to 4800 μM in case of CuO NP and even more pronounced to more than 70-fold from 300 μM up to 22,000 μM in case of CuO MP. These findings demonstrate the importance of removing the membranes since otherwise the copper uptake would be considerably overestimated.

In A549 cells the copper uptake into cytoplasm and nucleus has been investigated previously; here, CuO NP and CuCl_2_ led to a concentration-dependent copper increase in the cytoplasm, even though less accumulation was seen when compared to BEAS-2B cells. In contrast, CuO MP led to higher, albeit highly variable cytoplasmic concentrations in A549 cells. With respect to the copper concentrations in the nucleus, effects of CuO NP and CuO MP were similar in A549 and BEAS-2B cells; in case of CuCl_2_, elevated copper levels were detected only in the former cell line, coincident with higher cytotoxicity [14].

### Gene expression analyses

The impact of CuO NP, CuO MP and CuCl_2_ on gene expression profiles related to genomic stability was investigated in BEAS-2B and A549 cells after 24 h incubation via a high-throughput RT-qPCR technique [24]. The main observations obtained in both cell lines sorted in distinct signalling pathways and cellular processes are described in the following sections.

#### Impact on genes related to copper uptake and (oxidative) stress response

**Fig. 3:**
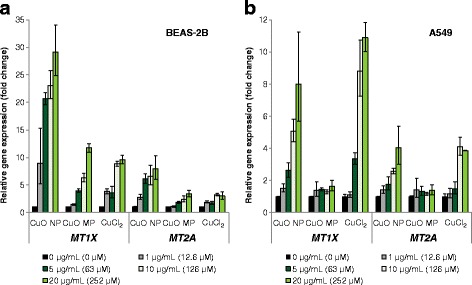
Impact of CuO NP, CuO MP and CuCl_2_ on gene expression of *MT1X* and *MT2A* related to copper uptake. BEAS-2B cells **(a)** or A549 cells **(b)** were treated with the different copper compounds for 24 h. Shown are linear fold changes of the relative gene expression from mean values of four determinations derived from two independent experiments ± SD. 20 μg/mL CuO are equal to 252 μM Cu^2+^. Please note the different scaling of the y-axis

The three copper compounds induced a pronounced dose-dependent up-regulation of the metallothionein genes *MT1X* and *MT2A* (Fig. 3). Strongest induction was evident by CuO NP in BEAS-2B cells (Fig. 3a). *MT1X* was transcriptionally enhanced to 9-fold at 1 μg/mL and increasing to 29-fold at 20 μg/mL, whereas mRNA levels of *MT2A* were elevated up to 8-fold. The expression of these genes was also induced by CuO MP and CuCl_2_, albeit to a lesser extent, reaching 12-fold (*MT1X*) and 3-fold (*MT2A*) increases in case of CuO MP and CuCl_2_, respectively. In A549 cells (Fig. 3b) the increase of transcript levels was less pronounced as compared to BEAS-2B cells; both genes were induced dose-dependently in case of CuO NP up to 11- and 4-fold, respectively, while CuO MP showed no effect. CuCl_2_ enhanced transcription to a similar extent as CuO NP; however, induction was restricted to higher concentrations exerting considerable cytotoxicity in this cell line [14].

**Fig. 4:**
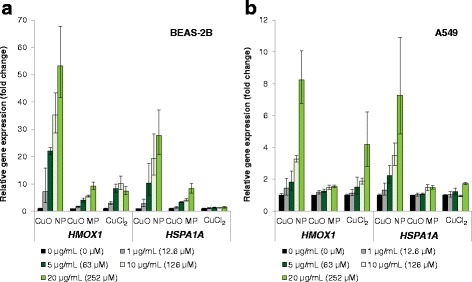
Impact of CuO NP, CuO MP and CuCl_2_ on gene expression of *HMOX1* and *HSPA1A* related to oxidative stress response. BEAS-2B cells **(a)** or A549 cells **(b)** were treated with the different copper compounds for 24 h. Shown are linear fold changes of the relative gene expression from mean values of four determinations derived from two independent experiments ± SD. 20 μg/mL CuO are equal to 252 μM Cu^2+^. Please note the different scaling of the y-axis

An induction of the oxidative stress response system at the transcriptional level was again most pronounced after treatment with CuO NP, with strongest effects in BEAS-2B cells (Fig. 4). Thus, in BEAS-2B cells expression of the ROS-inducible gene *HMOX1* was up-regulated concentration-dependently up to 53-fold by CuO NP and up to 10-fold by the other copper compounds. Transcription of the heat-shock sensitive *HSPA1A* was increased up to 28-fold or up to 8-fold by CuO NP or CuO MP, respectively, whereas no effects were observed after treatment with CuCl_2_ (Fig. 4a). In A549 cells *HMOX1* and *HSPA1A* were almost equally enhanced by CuO NP on the transcriptional level to a maximum of 8-fold or 7-fold, respectively. CuCl_2_ and CuO MP induced no changes in gene expression, except for *HMOX1* in case of CuCl_2_ at the highest concentration (Fig. 4b).

**Fig. 5:**
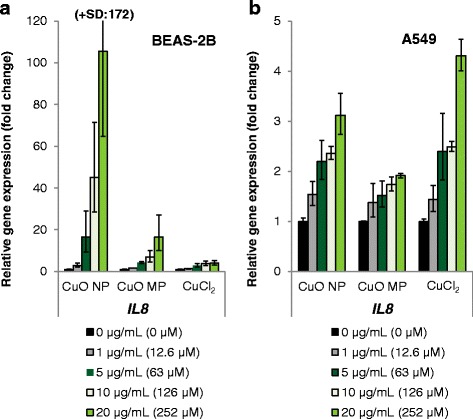
Impact of CuO NP, CuO MP and CuCl_2_ on gene expression of inflammation marker *IL8*. BEAS-2B cells **(a)** or A549 cells **(b)** were treated with the different copper compounds for 24 h. Shown are linear fold changes of the relative gene expression from mean values of four determinations derived from two independent experiments ± SD. 20 μg/mL CuO are equal to 252 μM Cu^2+^. Please note the different scaling of the y-axis

Induction of inflammation marker *IL8* was also far more pronounced in BEAS-2B cells as compared to A459 cells and most evident after treatment with CuO NP (Fig. 5). CuO NP caused a dose-dependent more than 100-fold increase in gene expression, while CuO MP and CuCl_2_ induced transcription only up to 16-fold or 4-fold, respectively. In A549 cells *IL8* expression was activated 3-fold (CuO NP), 4-fold (CuCl_2_) or 2-fold (CuO MP); again CuCl_2_ displayed a slightly stronger effect than CuO NP, but restricted to cytotoxic concentrations.

#### Impact on genes related to anti-oxidative defense, cell cycle regulation, apoptosis and DNA damage response and repair

**Fig. 6:**
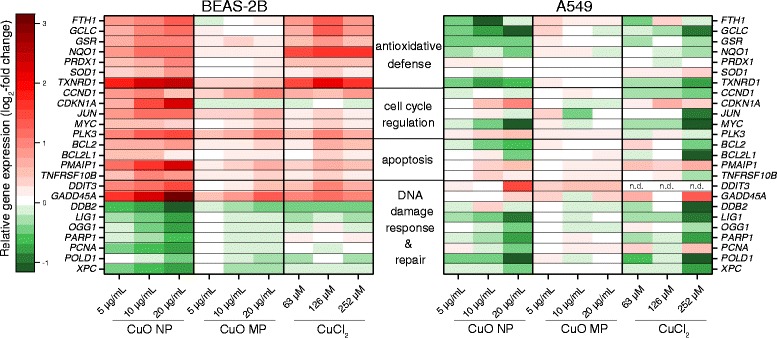
Impact of CuO NP, CuO MP and CuCl_2_ on gene expression related to anti-oxidative defense, cell cycle regulation, apoptosis and DNA damage response and repair. BEAS-2B cells or A549 cells were treated with the different copper compounds for 24 h. Shown are log_2_-fold changes of the relative gene expression in a heat map view from mean values of four determinations derived from two independent experiments (value of control = 0). 20 μg/mL CuO are equal to 252 μM Cu^2+^. n.d.: not determined

The impact of CuO NP, CuO MP and CuCl_2_ on genes associated to the anti-oxidant defense system, cell cycle regulation and proliferation, apoptosis as well as DNA damage response and repair in both cell lines is summarized in a heat map view (Fig. 6). In contrast to the gene expression results shown above, changes in gene expression are presented as log_2_-fold changes with a value of 0 for the untreated control since a repression of transcription frequently observed within these signalling groups became clearer within this depiction.

Most strikingly, strong modulations were observed in case of CuO NP already evident at the lowest concentration in BEAS-2B cells. Thus, a strictly dose-dependent induction of genes coding for anti-oxidative enzymes like *FTH1*, *GCLC, GSR*, *NQO1*, *PRDX1, SOD1* and *TXNRD1,* as well as of the proliferation-associated *JUN, MYC, PLK3* genes along with the induction of cell cycle inhibitor gene *CDKN1A* was observed in case of CuO NP in this cell line. Likewise, expression levels of the pro-apoptotic genes *PMAIP1* and *TNFRSF10B* were induced together with enhanced transcription of the anti-apoptotic *BCL2* and *BCL2L1* genes*,* affecting both the extrinsic and the intrinsic pathway. Concerning the DNA damage response, transcription of the damage inducible genes *DDIT3* and *GADD45A* was increased. However, the expression of specific DNA repair genes like the BER-associated *LIG1, OGG1* and *PARP1*, the NER-associated *DDB2* and *XPC* genes as well as *PCNA* was repressed by CuO NP in a concentration-dependent manner. With respect to CuO MP almost no modulation of the above mentioned genes was observed in BEAS-2B cells; by tendency altered transcript levels were at most restricted to the highest concentration. Intermediate effects occurred in case of CuCl_2_. Some anti-oxidative defense-associated as well as apoptosis related genes were induced together with a moderate induction of *DDIT3* and *GADD45A*, while in case of the DNA repair genes only by tendency repressed transcript levels were observed. Most notably, no dose-dependency was obvious in case of CuCl_2_, since most relevant changes occurred in case of 126 μM and not 252 μM.

In A549 cells a completely different gene expression pattern was apparent. In case of CuO NP mainly repressed transcript levels were observed, especially with respect to the anti-oxidative defense-associated genes. Only genes coding for the cell cycle inhibitor p21 (*CDKN1A*), the pro-apoptotic Noxa (*PMAIP1*) and the DNA damage inducible DDIT3 (*DDIT3*) were transcriptionally slightly enhanced after treatment with CuO NP. With respect to pro-apoptotic signalling, effects were restricted to the intrinsic pathway (*PMAIP1),* along with slightly repressed mRNA levels of the anti-apoptotic genes *BCL2* and *BCL2L1.* No effects at all were observed in case of CuO MP. CuCl_2_ displayed a gene expression modulation largely comparable to CuO NP; however, in contrast to CuO NP, pronounced effects were restricted to the highest, already cytotoxic concentration.

### Intracellular GSH content

**Fig. 7:**
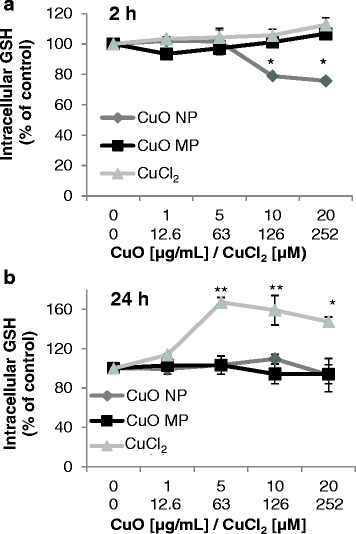
Impact of CuO NP, CuO MP and CuCl_2_ on the intracellular GSH level. BEAS-2B cells were treated for 2 h **(a)** or 24 h **(b)**, respectively, with the different copper compounds. Shown are mean values of four determinations derived from two independent experiments ± SD. Statistically significant different from control: *p ≤ 0.05*,* **p ≤ 0.01 (ANOVA-Dunnett’s T test). 20 μg/mL CuO are equal to 252 μM Cu^2+^

The induction of oxidative stress was investigated via modulations of the intracellular GSH levels after treatment with CuO NP, CuO MP and CuCl_2_ in BEAS-2B cells according to the method established by Tietze [28]. CuO NP led to slightly reduced intracellular GSH levels to 80% of control beginning at 10 μg/mL after 2 h (Fig. 7a **)**. In contrast, CuO MP or CuCl_2_ displayed no alterations at this time point. After a 24 h treatment period no changes were observed in case of the particulate copper compounds; however, CuCl_2_ displayed a pronounced increase up to 150–170% in the concentration range of 63–252 μM (Fig. 7b **)**.

### Nrf2 activation

**Fig. 8:**
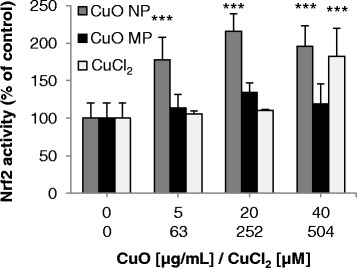
Impact of CuO NP, CuO MP and CuCl_2_ on Nrf2 activity. HeLa S3 cells were treated for 5 h with the different copper compounds. Shown are mean values of three independent experiments + SD. Statistically significant different from control: ***p ≤ 0.001 (ANOVA-Dunnett’s T test). 20 μg/mL CuO are equal to 252 μM Cu^2+^

Furthermore, a potential activation of the redox-sensitive transcription factor Nrf2 was examined. Since nuclear Nrf2 levels were too low for quantification in BEAS-2B cells when using a reasonable cell number and A549 cells on the other hand exert a constitutively activated Nrf2 due to a dysfunction of its negative inhibitor Keap1 [29], these investigations were performed in HeLa S3 cells as a model. Experiments were accomplished by a TransAm Nrf2 ELISA assay, quantifying the binding capacity of nuclear located Nrf2 to the ARE consensus binding site (Fig. 8). Considering the time point of most pronounced activation of Nrf2 by the positive control sulforaphane (data not shown), cells were treated for 5 h with the respective copper compounds applying a concentration range from 5 to 40 μg/mL in case of CuO NP and CuO MP, or 63–504 μM in case of CuCl_2_. The concentrations were selected based on cytotoxicity of the copper compounds in this cell line as previously published [14]. While CuO MP were not cytotoxic within the entire concentration range applied, CuO NP and CuCl_2_ exerted strong cytotoxicity starting at 5 μg/mL or 126 μM, respectively. Furthermore, uptake studies with the copper compounds were performed in this cell line to assess the suitability as a model for Nrf2 activation (Additional file 2). Here, a concentration-dependent increase of intracellular copper levels reaching more than 2.5 mM was observed in case of CuO NP after 8 h and 24 h; copper accumulation was even more pronounced as compared to BEAS-2B cells. In contrast, copper levels were not altered by CuCl_2_ or CuO MP except for the high concentration of 50 μg/mL. Consequently, since cytotoxicity as well as copper uptake were generally comparable to BEAS-2B and A549 cells the use of HeLa S3 cells as a model was acceptable. Only CuO NP induced a strong activation of Nrf2 of 180–210% within the entire concentration range, whereas no activation of the transcription factor was observed in case of CuO MP; CuCl_2_-induced activation was restricted to the highest, cytotoxic concentration of 504 μM.

### Cell cycle distribution and apoptosis

The impact of CuO NP, CuO MP and CuCl_2_ on cell cycle distribution, apoptosis and necrosis at different times was investigated via flow cytometry in BEAS-2B cells. Cell cycle analyses were performed using the DNA intercalating fluorescent dye DAPI and the distinction between viable, apoptotic and necrotic cells took place using annexin V-FITC and propidium iodide (PI) staining.

**Fig. 9:**
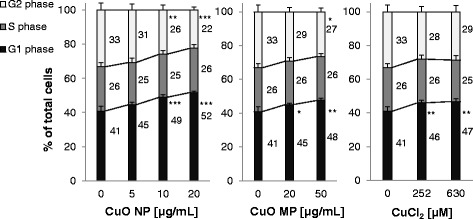
Impact of CuO NP, CuO MP and CuCl_2_ on cell cycle distribution. BEAS-2B cells were treated with the different copper compounds for 8 h. Shown are mean values of four determinations derived from two independent experiments + SD. Statistically significant different from control: *p ≤ 0.05*,* **p ≤ 0.01*,* ***p ≤ 0.001 (ANOVA-Dunnett’s T test). The numbers right to each bar indicate the fraction of cells in the respective phase. Please note the different concentrations used for the respective copper compound. 20 μg/mL CuO are equal to 252 μM Cu^2+^

The effect on cell cycle distribution was most evident after 8 h (Fig. 9). Thus, CuO NP led to a dose-dependent increase of cells in G0/G1 phase from 40% in the control up to more than 50% in case of 20 μg/mL. The amount of S phase cells did not alter, but cells in G2/M phase decreased accordingly. CuO MP and CuCl_2_ affected the cell cycle distribution in a similar manner. However, the modulation occurred to a lesser extent with relevant effects restricted to high concentrations starting at 20 μg/mL or 252 μM, respectively. After 24 h no alteration within cell cycle distribution was persistent (Additional file 3).

**Fig. 10:**
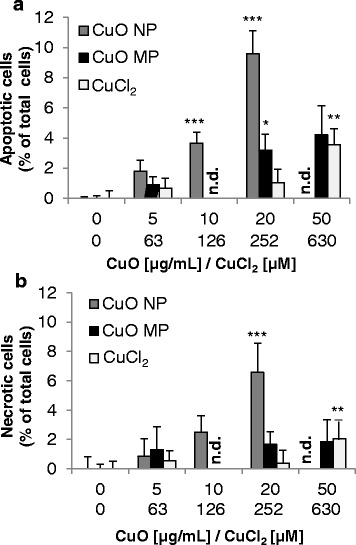
Induction of apoptosis **(a)** and necrosis **(b)** after treatment with CuO NP, CuO MP and CuCl_2_. BEAS-2B cells were treated with the different copper compounds for 48 h. Shown are mean values of four determinations derived from two independent experiments + SD. Statistically significant different from control: *p ≤ 0.05*,* **p ≤ 0.01*,* ***p ≤ 0.001 (ANOVA-Dunnett’s T test). 20 μg/mL CuO are equal to 252 μM Cu^2+^. n.d.: not determined

With respect to cell death induction, only weak effects were observed after 24 h (Additional file 4), whereas a distinct modulation was obvious after 48 h (Fig. 10). Thus, incubation with CuO NP concentration-dependently enhanced the amount of apoptotic cells to a maximum of nearly 10%, whereas CuO MP and CuCl_2_ displayed a maximum of 4% apoptotic cells. The amount of necrotic cells was close to 7% in case of CuO NP and 2% in case of CuO MP and CuCl_2._ Altogether, cell death was mediated about equally by apoptosis and necrosis.

## Discussion

Within the present study, cytotoxicity, cellular uptake and intracellular copper accumulation after treatment with CuO NP as compared to CuO MP and water soluble CuCl_2_ were investigated in the immortalized human bronchial epithelial cell line BEAS-2B and compared to findings in the adenocarcinoma cell line A549 previously performed in our working group [14]. Moreover, the impact on cellular signalling pathways involved in the maintenance of genomic stability was analysed in both cell lines by systematic, concentration-dependent gene expression profiling via a high-throughput RT-qPCR approach. Some observations on mRNA level were pursued on the functional level by investigating induction of apoptosis, oxidative stress as well as modulation of cell cycle distribution in BEAS-2B cells. Main outcomes were a distinct accumulation of copper in the soluble fractions of the cytoplasm and the nucleus after treatment with CuO NP, leading to pronounced changes in gene expression related to metallothionein induction, oxidative stress response, DNA damage response, cell cycle regulation and apoptosis. Far less effects were observed in case of CuO MP. Interestingly, CuCl_2_ led to a copper accumulation in the cytoplasm, but not in the nucleus, and cellular effects were mainly restricted to cytotoxic concentrations with disturbed copper homeostasis.

The stronger cytotoxicity of CuO NP when compared to CuO MP or CuCl_2_ confirmed previous observations on the cytotoxicity of differently sized CuO particles and water-soluble copper compounds in lung-associated cell models [12, 13, 15, 30]. Additionally, CuO NP exerted more pronounced cytotoxic effects in BEAS-2B than in A549 cells, even though in the latter cell line the more sensitive colony forming ability was applied [14]. The higher sensitivity of BEAS-2B cells towards CuO NP as compared to A549 cells was also reported by Cronholm and colleagues [12]. This may be due to the higher GSH content of A549 compared to BEAS-2B cells [31–33]. However, interestingly, cytotoxicity of CuCl_2_ was higher in the former cell line.

As stated in the introduction, the major mechanism proposed for the high toxicity of CuO NP consists in the so-called “Trojan horse type mechanism” [17], comprising the endocytotic uptake of the particles, the solubilisation in lysosomes and the subsequent intracellular copper ion release. Endocytosis of particulate CuO has been shown in several studies [20–23]. While electron microscopy enables the detection of particles inside the cell and thus can demonstrate particle uptake, it does not allow an exact quantification of endocytosed particles. Considering the whole cell, a more efficient uptake of CuO NP in BEAS-2B cells as compared to A549 cells was already shown previously, determined by AAS [12]. However, since particles tend to bind to cell membranes and cannot be removed completely by cell washing, quantification of copper content of treated cells by AAS or ICP-MS does not allow to discriminate between copper within particles attached to the cells and deliberated copper ions inside the cell. Within the present study, therefore, copper was quantified by AAS, measuring total copper contents in cells and additionally by applying a specific two step lysis procedure. With respect to the latter, an approach has been chosen where the plasma membrane was lysed in the first step, yielding the soluble cytoplasmic fraction and cell nuclei. These were lysed in the second step, yielding the soluble fraction of the nuclei. Concerning the intracellular distribution, a strictly concentration-dependent copper accumulation up to millimolar concentrations both in the cytoplasm and even more pronounced in the nucleus was shown for the first time for CuO NP in BEAS-2B cells, exceeding again the values observed previously in A549 cells [14]. While the high copper contents in the cytoplasm could result both from remaining nanoparticles not yet completely solubilized in the lysosomes as well as from released copper ions, the even higher copper concentration in the nucleus strongly indicates the release of copper ions. These findings are in accordance with the far higher solubility of CuO NP in the acidic artificial lysosomal fluid (ALF) as compared to water or cell culture media [14]. CuO MP were taken up to a much lesser extent, but also in a concentration-dependent manner, yielding lower concentrations also in the cytoplasm and in the nucleus. Comparison to whole cell copper contents without removal of the outer membrane after particle treatment performed within this study demonstrated that overestimation of cellular copper levels would have been 3.5-fold in case of CuO NP and even 70-fold in case of CuO MP. In contrast to the particulate copper compounds, incubation with CuCl_2_ provoked comparatively slightly elevated copper levels in BEAS-2B cells. Especially in the nucleus almost no increase was observed, indicating a tight homeostatic control due to regulated uptake of copper ions. In contrast, higher nuclear copper levels have been observed in A549 cells [14], which may be explained by the more pronounced cytotoxicity in the latter cell line, leading to uncontrolled copper influx. Consequently, endocytotic uptake appears to circumvent copper homeostasis even at non-cytotoxic concentrations, while in case of water soluble copper compounds impaired homeostasis is restricted to cytotoxic concentrations. Altogether, the intracellular copper accumulation may be most decisive for the toxicity of CuO NP.

The systematic gene expression profiling via high-throughput RT-qPCR displayed a concentration-dependent impact of CuO NP on genes related to uptake and genomic stability. The extent of modulation of the uptake-related *MT1X* and *MT2A* genes, which are up-regulated in response to copper ions via the metal responsive transcription factor MTF-1 [34, 35], reflected the dose-dependent intracellular copper ion increase, exerting distinct differences among the cell lines as well as between the copper compounds. Thus, induction of *MT1X* and *MT2A* by CuO NP was very distinct in case of all investigated concentrations, higher than for CuO MP and CuCl_2_ as well as more pronounced in BEAS-2B cells as compared to A549 cells. Since metallothionein gene induction is mediated by copper ions and not the particulate compounds this is one clear indication of the marked intracellular release of copper ions by the particles.

Moreover, the up-regulation of oxidative and inflammatory response genes was more distinct in BEAS-2B than in A549 cells and again most pronounced in case of CuO NP; the concentration-dependent effects highly corresponded to the intracellular copper accumulation. The induction of the *HMOX1* und *HSPA1A* genes is mediated via different redox-sensitive transcription factors like Nrf2, HSF-1 and AP-1 [36–38] and can be regarded as a model transcriptional response to the extent of ROS generation and activation of the respective transcription factors by the copper compounds. *IL8* gene displayed highest induction of all investigated genes by CuO NP in BEAS-2B cells. This gene is known to be highly inducible by NF-ΚB and AP-1 in response to pro-inflammatory cytokines or cellular stress [39]. A pro-inflammatory response via IL-8 release coincident with an activation of AP-1 and NF-ΚB was further reported in case of CuO NP in A549 cells [40] and was also observed in A549 and HBEC cells using an in vitro air-liquid interface exposure system [41]. An induction of *IL-8* was also described in response to TiO_2_ NP via NF-ΚB and AP-1 activation in different lung cell lines, indicating that oxidative stress may not be the only reason to induce respective signalling pathways [42].

The induction of genes of the anti-oxidant defense system, mainly regulated via Nrf2 [43], by CuO NP and to a lesser extent by CuCl_2_ was restricted to BEAS-2B cells, whereas the transcript levels were not altered or even repressed in A549 cells. These differences between the cell lines may be explained by lower levels of intracellular glutathione in BEAS-2B cells as compared to A549 cells [31–33, 44, 45] as well as by a persistent deregulation of the expression of those anti-oxidative genes due to constitutively active Nrf2 in A549 cells [29]. The proposed activation of Nrf2 was confirmed in case of CuO NP for all applied concentrations and in case of CuCl_2_ in high cytotoxic doses in HeLa S3 cells. These cells were chosen since they exert lower GSH levels as compared to A549 cells [46] and are thus in this respect comparable to BEAS-2B cells, and no dysfunctional regulation of Nrf2 has been reported. Since HeLa S3 cells are derived from cervical adenocarcinoma, results obtained in this cell line are not directly transferable to the lung cell models; however, the results show that Nrf2 is in principle activated by CuO NP and CuCl_2_. In support of using HeLa S3 cells as a model for this specific question, cytotoxicity as well as copper accumulation occurred to a comparable extent as in BEAS-2B and A549 cells. The induction of oxidative stress on the functional level was further analysed via changes in intracellular GSH levels in BEAS-2B cells. Accordingly, GSH depletion to 80% of control was limited to CuO NP after 2 h treatment; in case of CuCl_2,_ after 24 h elevated intracellular GSH levels were observed, indicative of up-regulation of GSH synthesis and/or GSH reductase activity. These results confirm previous studies reporting oxidative stress induction, measured by enhanced ROS levels, changes in GSH and GSSG, lipid peroxidation or modulated activities of anti-oxidant enzymes by CuO NP at different time points [9, 47, 48].

With respect to genes associated with cell growth, cell cycle regulation and apoptosis, increased mRNA levels of the proto-oncogene *JUN* as well as the induction of cell cycle inhibitor gene *CDKN1A* were restricted to CuO NP in BEAS-2B cells. The positively auto-regulated *JUN* [49–51] together with the induced *IL8* and *HMOX1* genes described above strongly indicates an activation of AP-1 by CuO NP. In accordance with *CDKN1A* induction, a cell cycle arrest in G0/G1 phase was observed after 8 h predominantly in case of CuO NP on the functional level. The induction of the pro-apoptotic genes *PMAIP1* und *TNFRSF10B* was restricted to CuO NP in BEAS-2B cells. Thus, genes coding for factors of both the intrinsic and the extrinsic cascade were activated in this cell line. However, the outcome concerning the intrinsic pathway was conflicting by the concurrent slight induction of genes of the anti-apoptotic Bcl-2 family. In contrast, in A549 cells only genes coding for the intrinsic pathway were modulated, indicating a weak, but clear pro-apoptotic signalling by a slight induction of *PMAIP1,* coincident with decreased anti-apoptotic *BCL2* and *BCL2L1* transcript levels. On the functional level, cell death mediated by CuO NP and to a lesser extent by CuO MP was necrotic and apoptotic, the latter slightly more pronounced. Consistent with the gene expression results the induction of apoptosis was restricted to CuO NP in BEAS-2B cells, first evident after 48 h treatment. In A549 cells previous analyses revealed the restriction of apoptosis to high concentrations of more than 20 μg/mL CuO NP [14].

Indicative for the induction of DNA damage, the DNA damage response inducible genes *DDIT3* and *GADD45A* [52–54] were strongly up-regulated by CuO NP in BEAS-2B cells, while only slight effects were observed in case of CuO MP and CuCl_2_. Induction of these genes was also evident in A549 cells, albeit to a lesser extent and restricted to higher concentrations. The results are in agreement with previous observations, demonstrating enhanced expression of *p38* and *p53*, indicative for DNA damage, and the induction of DNA damage inducible genes of the GADD family by CuO NP [19, 55]. The activation of the DNA damage response is consistent with the induction of DNA strand breaks by CuO NP measured via comet assay or alkaline unwinding [12, 14]. In addition to genotoxicity, copper ions appear to inactivate DNA repair processes. Thus, genes coding for specific proteins in DNA repair pathways such as BER and NER were down-regulated by CuO NP in BEAS-2B cells and by both CuO NP and CuCl_2_ in A549 cells. In addition, interactions of copper with distinct DNA repair proteins have been described on the functional level, e.g. XPA in NER and hOGG1 and PARP-1 in BER [56–58]. The inhibition of PARP-1 could further be confirmed by CuO NP and CuO MP [14]. Despite the DNA damage signalling, no activation of p53 appears to occur. Thus, no induction of its inhibitor *MDM2*, which is transcriptionally regulated by activated p53 [59], was observed (data not shown). In BEAS-2B cells p53 is inactivated as a consequence of the immortalization process of this cell line [25]. In A549 cells the missing activation was surprising since p53 is usually stabilized upon DNA damage, regulating cell cycle arrest and apoptosis. However, in accordance with our findings Hanagata and co-workers reported a minor role of p53 within gene expression induced by CuO NP [55].

As underlying mechanism of the observed oxidative and genotoxic stress response the increased generation of ROS due to elevated levels of redox-active copper ions can be assumed [12, 13, 15, 19, 48]. Further interactions leading for example to repressed transcript levels of DNA repair genes could include a decrease in mRNA stability, other post-translational mechanisms leading to diminished protein levels or the direct interaction with the respective transcription factors. Thus, due to the high thiol affinity and redox-activity of copper ions redox-sensitive cysteines inside or outside of zinc binding structures in transcription factors may display important targets of copper toxicity [60].

## Conclusions

Within the present study, we confirmed the pronounced cytotoxicity of CuO NP as compared to microsize CuO particles or water soluble CuCl_2_ in human lung cell lines. A CuO NP-mediated copper overload in the cytoplasm and especially in the nucleus was shown to be of major importance for the cytotoxic and genotoxic potential as well as for dose-dependent transcriptional changes of genes related to copper overload, (oxidative) stress response, DNA damage response, cell growth and cell cycle regulation and apoptosis. The high-throughput RT-qPCR approach provided insight into the mode of action of CuO NP and revealed relevant differences not only between the copper compounds, but also between the different cell lines. Importantly, it allowed the identification of dose-dependency; thus, while CuO NP exerted effects already at low, non-cytotoxic concentrations, changes in gene expression mediated by water soluble CuCl_2_ were restricted to cytotoxic concentrations with disturbed copper homeostasis. One underlying mechanism appears to be increased intracellular amounts of “free” reactive copper ions, consistent with the “Trojan horse type mechanism”, which may lead to elevated levels of ROS, but also direct oxidation of redox-sensitive transcription factors such as critical cysteines in transcription factors. However, in addition to intracellular copper ion release, interactions of the CuO particles with the outer cell membrane cannot be completely excluded to contribute to the observed effects. Also, within this study, comparisons were made based on the copper content of the different compounds. Nevertheless, the impact of different surface areas of the nano- and microsized particles should be addressed. Finally, whether or not similar mechanisms account for the toxicity of other metal-based nanoparticles needs to be addressed on a case-to-case basis. These issues will be further investigated.

## Methods

### Materials

Chemicals, including agarose, salts, glycerol, leupeptine, phenylmethanesulfonyl-fluoride (PMSF), bovine serum albumin, acids, snap-on lid glasses and stirring bars were obtained from Carl Roth GmbH (Karlsruhe, Germany). Copper chloride was purchased in high purity (>99.9%) from Sigma-Aldrich Chemie GmbH (Steinheim, Germany). All PCR consumables including PCR tubes, strips and reaction tubes and tubules as well as cell culture dishes and flasks were obtained from Sarstedt (Nuembrecht, Germany). The primer pairs were synthesized by Eurofins (Ebersberg, Germany) or Fluidigm (San Francisco, USA). DMEM, trypsin, amphotericin B, trypsin inhibitor from *Glycine max* (soybean) (SBTI) and penicillin-streptomycin solutions are products of Sigma-Aldrich. Fetal calf serum (FCS) and LHC-9 media are products of Invitrogen GmbH (Darmstadt, Germany). Human fibronectin was obtained from Biopur (Reinach, Switzerland) and collagen from Roche (Mannheim, Germany). DNA suspension buffer, PCR certified water and TE buffer were obtained from Teknova (Hollister, USA). 2X Assay Loading Reagent and 20X DNA Binding Dye Sample Loading Reagent were purchased from Fluidigm (San Francisco, USA). Bio-Rad (Munich, Germany) provided the 2X SsoFast™ EvaGreen® Supermix with Low ROX and the 2X SYBR Green Supermix. The 2X TaqMan® PreAmp Master Mix was obtained from Applied Biosystems (Darmstadt, Germany) and the exonuclease I from New England Biolabs (Frankfurt am Main, Germany).

### Particle characteristics

CuO NP (#544868, Lot #MKAA0633) and CuO MP (#208841, Lot #MKAA1788) were purchased from Sigma-Aldrich Chemie GmbH (Steinheim, Germany). A detailed particle characterization using DLS with respect to size, scanning electron microscopy (SEM) for size and morphology, BET for surface area, ZP for surface charge, ICP-MS, EDX and oxygen analysis for purity and composition as well as X-ray diffraction (XRD) for crystallinity was described previously. Additionally, the impact on pH in relevant media, endotoxin content and solubility in different fluid models like H_2_O, DMEM, DMEM/10% FCS, PBS, AAF and ALF was investigated [14].

### Particle and CuCl_2_ incubation suspensions and dilutions

Fresh CuO NP, CuO MP suspensions and CuCl_2_ dilutions were prepared for each experiment. Particles, received as dry powder, were aliquoted by weighing into 1.5 mL polystyrene reaction tubes. Stock solutions of 1 mg/mL CuO were prepared into an endotoxin-free snap-on glass containing a stirring bar in DMEM containing 10% FCS. Dilutions in the range of 1–50 μg/mL were prepared by adding aliquots of the stirring stock solution into snap-on lid glasses filled with adequate volumes of fresh medium. Stirring took place at 900 rpm and room temperature on a multiphase stirrer (Variomag Poly, Carl Roth GmbH, Karlsruhe, Germany). CuCl_2_ was dissolved in bidistilled H_2_O (100 mM) and sterile-filtered. Dilutions in the range of 12.6–630 μM corresponding to the identical mass doses of copper per mL of the particulate CuO were prepared from the stock solution by dilution in adequate amounts of DMEM containing 10% FCS directly before incubation. 1 μg/mL CuO is equal to 0.2 μg/cm^2^ CuO and 12.6 μM Cu^2+^ in case of complete dissolution.

### Cell culture and incubation

The adherent human adenocarcinoma cell line A549 (ATCC CCL-185) and human cervix adenocarcinoma cell line HeLa S3 (ATCC CCL-2.2) were obtained from ATCC and cultured as monolayer in DMEM containing 10% FCS, 100 U/mL penicillin and 100 mg/mL streptomycin. Human lung bronchial epithelial BEAS-2B cells (ATCC CRL-9609), immortalized with SV40 large T-antigen, were kindly provided by Dr. Carsten Weiss (Karlsruhe Institute of Technology, Karlsruhe, Germany). They were grown as monolayers in coated cell culture dishes (10 μg/mL human fibronectin, 30 μg/mL collagen and 10 μg/mL bovine serum albumin in PBS) in LHC-9 medium containing 2.5 μg/mL amphotericin B. Cells were incubated at 37 °C in a humidified atmosphere of 5% CO_2_ in air. For all experiments cells were seeded at a density of 16.600 cells/cm^2^. After 24 h of cultivation in case of A549 and HeLa S3 cells and 48 h in case of BEAS-2B cells the supernatant from the logarithmically growing cells was removed and replaced by the particle and CuCl_2_ incubation suspensions or dilutions (0.2 mL/cm^2^) in DMEM containing 10% FCS independent from the respective cell culture medium to ensure comparable incubation conditions for all applied cell lines.

### Cell number

Logarithmically growing BEAS-2B cells were incubated for 24 h with 1–50 μg/mL CuO NP or CuO MP or 12.6–630 μM CuCl_2_, respectively, trypsinized and collected in DMEM containing 10% FCS. Cell number was determined via Casy® cell counter (OLS OMNI Life Science GmbH & Co. KG, Bremen, Germany).

### Cellular uptake and intracellular distribution

Logarithmically growing BEAS-2B cells were treated with different concentrations of CuO NP, CuO MP and CuCl_2_ for 24 h in DMEM containing 10% FCS. The cells were trypsinized, collected in ice-cold PBS containing 10% FCS, washed twice with PBS and counted via Casy® cell counter for cell number and cell volume. For determination of copper in the soluble fraction of the whole cells, they were lysed in RIPA buffer (0.01 M Tris pH 7.6, 0.15 M NaCl, 0.001 M EDTA, 1% (*v*/v) Trition-X 100, 1% (*v*/v) DOC, 0.01% SDS, 0.001 M PMSF, 1 x protease-inhibitor mixture) for 30 min before centrifugation at 14.000 x g (1 h, 4 °C). The supernatant contained the soluble fraction and was used for graphite furnace atomic absorption spectrometry (GF-AAS). Soluble cytoplasmic and nuclear fractions were prepared using a method described previously [14, 27]. Briefly, cells were allowed to swell in cell lysis buffer (0.01 M HEPES pH 7.9, 0.01 M KCl, 0.0015 M MgCl_2_, 0.3 M saccharose, 0.0005 M dithiothreitol (DTT), 0.0006 M PMSF, 0.0065 mM Leupeptine) for 15 min before the addition of 25 μL 10% (*v*/v) IGEPAL CA 630 (Fluka) in H_2_O for cell lysis. The mixture was vortexed for 10 s and the nuclei pelleted at 1500 x g (15 min, 4 °C). The supernatant contained the soluble cytoplasmic fraction and was used for GF-AAS. The nuclei-containing pellet was washed twice with cell lysis buffer. Subsequently the volume and number of the nuclei was determined via Casy® cell counter and the soluble content was extracted by treatment with nuclear lysis buffer (0.01 M HEPES pH 7.9, 0.4 M KCl, 0.0015 M MgCl_2_, 15% (*w*/*v*) glycerol, 0.0005 M DTT, 0.0006 PMSF, 0.0065 mM Leupeptine) for 30 min on ice after mixing and subsequent centrifugation at 10.000 x g (30 min, 4 °C). The supernatant contained the soluble nuclear fraction and was used for GF-AAS. All cellular soluble fractions were evaporated at 95 °C, incubated with ashing mixture, 65% HNO_3_/30% H_2_O_2_ (1/1), evaporated at 95 °C again and resolved in water. Copper content was determined using GF-AAS (Perkin Elmer Atomic Absorption Spectrometer PinAAcle 900 T). Recovery rates were determined in the respective matrix using AAS elemental standard solutions (Carl Roth AG) and reached 101% ± 6% (external standard), 99% ± 4% (total fraction), 96% ± 6% (cytoplasmic fraction) and 99% ± 4% (nuclear fraction). Copper content was normalized to cell number and volume.

### Gene expression analyses

Gene expression analyses via high-throughput RT-qPCR with Fluidigm dynamic arrays on the BioMark™ System were performed as described previously [24]. Briefly, 0.35–1 × 10^6^ logarithmically growing A549 or BEAS-2B cells were treated with different concentrations of CuO NP, CuO MP and CuCl_2_ for 24 h in DMEM containing 10% FCS. After incubation, cells were trypsinized, resuspended in ice-cold PBS containing 10% FCS, collected by centrifugation, washed with ice-cold PBS and collected again by a second centrifugation step. Total RNA was isolated with MN NucleoSpin® RNA Plus KIT (Macherey-Nagel) according to the manufacturer’s instructions and quantified. 1 μg of total RNA was reverse transcribed in duplicate per sample into first-strand complementary DNA (cDNA) using qScript™ cDNA Synthesis Kit (Quanta) according to the manufacturer’s instructions. During high-throughput qPCR all pipetting steps were carried out in a separate room under decontaminated and sterile conditions in a PCR Workstation Pro (Peqlab, Erlangen, Germany). Before qPCR, specific target amplification (STA) and exonuclease I (*E. coli*) treatment had to be performed. A total of 5 μL STA mix was prepared containing 2.5 μL 2X TaqMan® PreAmp Master Mix, 0.5 μL of the 500 nM pooled primer mixture, 0.75 μL PCR certified water and 1.25 μL cDNA per reaction. A PCR certified water control (NTC-STA) and a non reverse-transcribed RNA control (NoRT) were included. STA was performed in a thermal cycler (T100, Bio-Rad Laboratories, Munich, Germany) using the following temperature program: 10 min at 95 °C followed by 12 cycles of 15 s at 95 °C and 4 min at 60 °C and a final holding temperature of 4 °C. Afterwards, 0.4 μL exonuclease I (Exo I) at 20 units/μL was diluted to 4 units/μL with 0.2 μL 10X Exonuclease I Reaction Buffer and 1.4 μL PCR certified water per reaction. 2 μL of the Exonuclease Reaction Mixture were added to the STA samples and digestion with Exo I at 4 units/μL was performed using the following temperature program: 40 min at 37 °C, 15 min at 80 °C and a final holding temperature at 4 °C. STA and Exo I-treated samples were diluted 5-fold with 18 μL TE buffer. For qPCR forward and reverse primers (100 μM) were diluted to 5 μM by adding 2.5 μL of each primer pair to 25 μL of 2X Assay Loading Reagent and 22.5 μL of DNA suspension buffer. The primer reaction mix was stored at −20 °C. For the sample mix, 2.25 μL of STA and Exo I-treated samples were mixed with 2.5 μL of 2X SsoFast™ EvaGreen® Supermix with Low ROX and 0.25 μL of 20X DNA Binding Dye Sample Loading Reagent. Preparation and loading of Fluidigm 96.96 Dynamic Array IFC (integrated fluidic circuit) was performed according to the manufacturer’s instructions. After priming, the chip was loaded with samples and the primer reaction mixes within 1 h to reduce the loss of the pressure within the chip. Thus, 5 μL of each primer reaction mix and each sample were pipetted into the respective inlets, avoiding the generation of air bubbles. Samples and primer reaction mixes were loaded into the chip by running the Load Mix (136×) script of the IFC Controller HX (Fluidigm, San Francisco, USA). After loading of the chip, potential dust particles were carefully removed from the surface of the chip using adhesive tape. The chip was transferred into the BioMark™ System (Fluidigm, San Francisco, USA) and qPCR and melting curve analysis were performed by running the following temperature program: 2400 s at 70 °C and 30 s at 60 °C, followed by a hot start for 60 s at 95 °C, 30 PCR cycles of 5 s at 96 °C for denaturation and 20 s at 60 °C for annealing and elongation. The melting curve analysis consisted of 3 s at 60 °C followed by heating up to 95 °C with a ramp rate of 1 °C/3 s. Data analysis and depiction was accomplished with the Fluidigm Real-Time PCR Analysis and with GenEx software. For normalization, five potential reference genes were available (*ACTB, B2M, GAPDH, GUSB,* and *HPRT1*). Finally, potential alterations of the transcript levels of the target genes under investigation were displayed as fold change compared to a control group by calculating relative quantities corresponding to the ΔΔC_q_ method [61].

### Quantification of intracellular glutathione

The quantification of intracellular glutathione levels was performed according to the method established by Tietze et al. [28]. Briefly, logarithmically growing BEAS-2B cells were incubated for 2 h or 24 h, respectively, with 1–20 μg/mL particulate CuO or 12.6–252 μM CuCl_2_, respectively, in DMEM containing 10% FCS. The cells were trypsinized, collected in ice-cold PBS containing 10% FCS, washed with PBS, counted via Casy® cell counter for cell number and cell volume and collected by centrifugation. 1 × 10^6^ cells were collected in KP buffer (0.1 M KH_2_PO_4_, 0.1 M K_2_HPO_4_, 1 mM EDTA, pH 7.4). Cells were lyzed by 2 freeze-and-thawing cycles followed by sonification, followed by acidification with sulphosalicyclic acid and vortexed. Cells were centrifuged at 16.000 rpm for 20 min at 4 °C prior to measuring total GSH in the supernatant by reducing oxidized GSH content using GR enzyme (4 U/mL) and NADPH (0.3 mM). Thereby, GSH reacts with 5,5′-dithiobis-2-nitrobenzoic acid (DTNB) to form 2-nitro-5-thiobenzoate (TNB). The change in TNB absorbance was measured at 412 nm using a plate reader (Tecan). Data were compared to GSH standard calibration curves and normalized to cell volume.

### Nrf2 activity

Logarithmically growing HeLa S3 cells were treated with different concentrations of CuO NP, CuO MP and CuCl_2_ for 5 h in DMEM containing 10% FCS. Nuclear extracts were prepared using a nuclear extract kit (Active Motif, Carlsbad, CA, USA) according to the manufacturer’s instructions. Thus, cells were washed with ice-cold PBS/phosphatase inhibitors, scraped into ice-cold PBS/phosphatase inhibitors buffer and collected by centrifugation. The cells were lyzed in hypotonic buffer and swelled on ice for 15 min. After addition of detergent and vortexing, cells were centrifuged at 14.000 x g for 30 s at 4 °C. The supernatant (containing the cytoplasmic fraction) was stored separately and the nuclear pellet was resuspended in Complete Lysis Buffer containing detergent, mixed and incubated for 30 min on a rocking platform at 150 rpm. Afterwards, the nuclear pellets were vortexed and centrifuged at 14.000 x g for 20 min at 4 °C. The supernatant containing the nuclear extract was used for the determination of Nrf2 activity after determining protein content according to the method established by Bradford [62]. Nrf2 activity was determined with the TransAM Nrf2 assay (Active Motif, Carlsbad, CA, USA) according to the manufacturer’s instructions. Nuclear extracts (5 μg) were incubated with ARE consensus site oligonucleotides (5′-GTCACAGTGACTCAGCAGAATCTG-3′) immobilized to 96-well plates. Bound protein levels were quantified by antibody specific to DNA-bound Nrf2 and visualized by colorimetric reaction catalysed by horseradish-peroxidase-conjugated secondary antibody. Resulting absorbance was measured at 405 nm with a reference wave length of 655 nm.

### Cell cycle analysis and cell death induction

0.35–1 × 10^6^ logarithmically growing BEAS-2B cells were treated with different concentrations of CuO NP, CuO MP and CuCl_2_ for 8, 24 or 48 h in DMEM containing 10% FCS. Cells were trypsinized, collected in fresh media, combined with the supernatant and centrifuged at 1300 rpm for 3 min at 4 °C. Respective parts of the cell suspension were used for cell death or cell cycle analysis. Concerning the latter, the pellet was resuspended in 1 mL ice-cold PBS before 3 mL ice-cold ethanol (96%) were added under vortexing, followed by fixation overnight at −20 °C. For flow cytometry analysis, the cells were collected by centrifugation, washed with 1 mL PBS and centrifuged again. The pellet was resuspended in 300 μL DAPI dye solution (CyStainDNA, Partec) and incubated for 30 min at 4 °C in the dark. For cell death analysis, the cell suspension was directly stained with a master mix (PI and annexin V-FITC in calcium buffer, pH 7.0) and incubated for 30 min at 4 °C in the dark. 10.000 or 30.000 cells per sample were analysed for cell death induction or DAPI intensity, respectively, via flow cytometry using a LSR Fortessa from Becton Dickinson. Excitation and emission detection of the applied fluorescence dyes were as follows: DAPI (405 nm - 450/50 nm), PI (488 nm – 695/40 nm) and annexin V-FITC (488 nm – 530/30 nm).

### Statistics

Differences between control and treated samples were analysed by one-way analysis of variance (ANOVA) followed by Dunnett’s T posthoc test.

## Additional files

Additional file 1: Cellular copper content after 24 h treatment with CuO NP or CuO MP with and without removal of the outer membrane in BEAS-2B cells. (PPTX 66 kb)

Additional file 2: Uptake studies on CuO NP, CuO MP and CuCl_2_ in HeLa S3 cells. (PPTX 71 kb)

Additional file 3: Supporting information on cell cycle distribution after 24 h treatment with different copper compounds in BEAS-2B cells. (PPTX 98 kb)

Additional file 4: Supporting information on cell death induction after 24 h treatment with different copper compounds in BEAS-2B cells. (PPTX 81 kb)

## Abbreviations

AAF: Artificial alveolar fluid
AIF: Apoptosis inducing factor
ALF: Artificial lysosomal fluid
AP-1: activator protein 1
BER: Base excision repair
BET: Brunauer-Emmett-Teller analysis
C_q_: Quantification cycle
CuCl_2_: Copper chloride
CuO MP: Copper oxide microparticles
CuO NP: Copper oxide nanoparticles
DLS: Dynamic light scattering
EDX: Energy-dispersive X-ray spectroscopy
GF-AAS: Graphite furnace atomic absorption spectrometry
GSH: Glutathione
GSSG: Glutathione disulfide
hOGG1: 8-oxoguanine DNA N-glycosylase 1
ICP-MS: Inductively coupled plasma mass spectrometry
Keap1: Kelch-Like ECH-Associated Protein 1
MDM2: Mouse Double Minute 2 protein
MTF-1: Metal-regulatory transcription factor 1
NER: Nucleotide excision repair
NF-ΚB: Nuclear factor ‘kappa-light-chain-enhancer’ of activated B-cells
Nrf2: Nuclear factor (erythroid-derived 2)-like 2
PARP-1: Poly(ADP-ribose)polymerase-1
PI: Propidium iodide
ROS: Reactive oxygen species
RT-qPCR: Reverse transcription quantitative polymerase chain reaction
SEM: Scanning electron microscopy
SOD: Superoxide dismutase
TEM: Transmission electron microscopy
XPA: Xeroderma Pigmentosum, Complementation Group A
ZP: Zeta potential
