# Supplementary material for: Comparison between micro- and nanosized copper oxide and water soluble copper chloride: interrelationship between intracellular copper concentrations, oxidative stress and DNA damage response in human lung cells
Source: Part Fibre Toxicol. 2017 Aug 1;14:28. doi: 10.1186/s12989-017-0209-1 (PMC5540434; doi:10.1186/s12989-017-0209-1)
Supplement: Supplementary file 1 — Cellular copper content after 24 h treatment with CuO NP or CuO MP with and without removal of the outer membrane in BEAS-2B cells. (PPTX 66 kb) [file 12989_2017_209_MOESM1_ESM.pptx]

## Slide 1
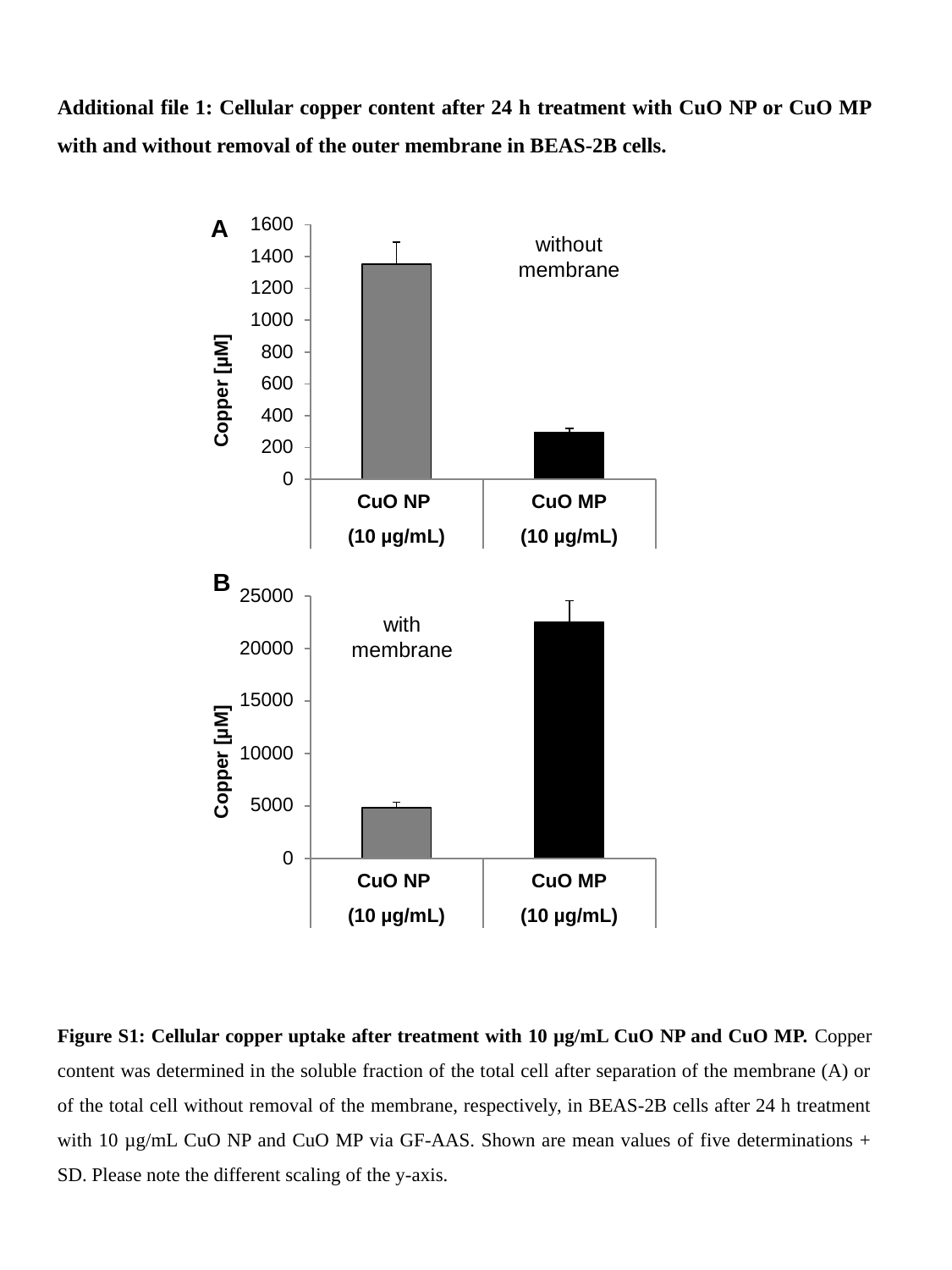

Additional file 1: Cellular copper content after 24 h treatment with CuO NP or CuO MP with and without removal of the outer membrane in BEAS-2B cells.
Figure S1: Cellular copper uptake after treatment with 10 µg/mL CuO NP and CuO MP. Copper content was determined in the soluble fraction of the total cell after separation of the membrane (A) or of the total cell without removal of the membrane, respectively, in BEAS-2B cells after 24 h treatment with 10 µg/mL CuO NP and CuO MP via GF-AAS. Shown are mean values of five determinations + SD. Please note the different scaling of the y-axis.
