# Supplementary material for: Comparison between micro- and nanosized copper oxide and water soluble copper chloride: interrelationship between intracellular copper concentrations, oxidative stress and DNA damage response in human lung cells
Source: Part Fibre Toxicol. 2017 Aug 1;14:28. doi: 10.1186/s12989-017-0209-1 (PMC5540434; doi:10.1186/s12989-017-0209-1)
Supplement: Supplementary file 2 — Uptake studies on CuO NP, CuO MP and CuCl2 in HeLa S3 cells. (PPTX 71 kb) [file 12989_2017_209_MOESM2_ESM.pptx]

## Slide 1
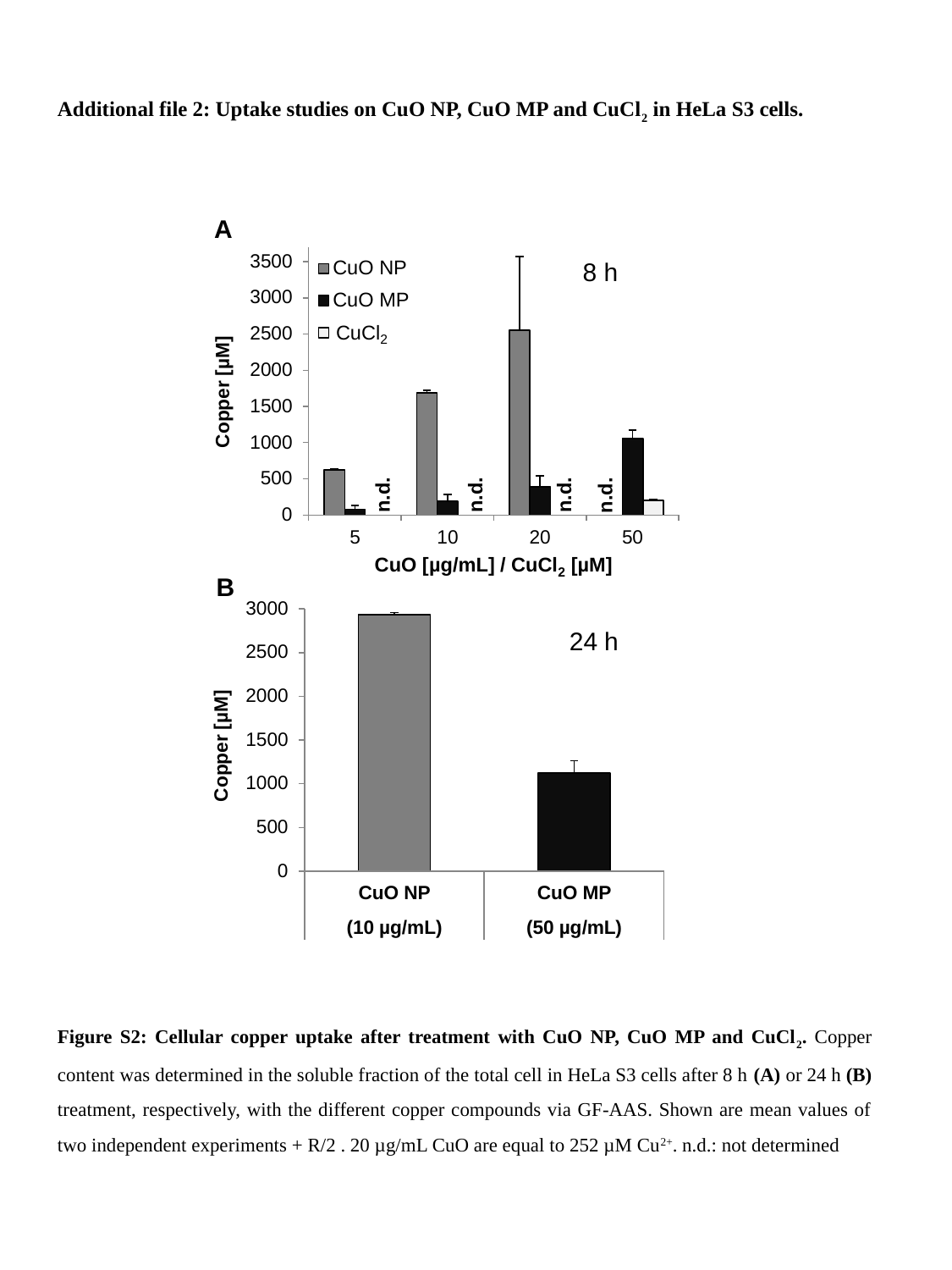

Additional file 2: Uptake studies on CuO NP, CuO MP and CuCl2 in HeLa S3 cells.
Figure S2: Cellular copper uptake after treatment with CuO NP, CuO MP and CuCl2. Copper content was determined in the soluble fraction of the total cell in HeLa S3 cells after 8 h (A) or 24 h (B) treatment, respectively, with the different copper compounds via GF-AAS. Shown are mean values of two independent experiments + R/2 . 20 µg/mL CuO are equal to 252 µM Cu2+. n.d.: not determined
