# Supplementary material for: Comparison between micro- and nanosized copper oxide and water soluble copper chloride: interrelationship between intracellular copper concentrations, oxidative stress and DNA damage response in human lung cells
Source: Part Fibre Toxicol. 2017 Aug 1;14:28. doi: 10.1186/s12989-017-0209-1 (PMC5540434; doi:10.1186/s12989-017-0209-1)
Supplement: Supplementary file 3 — Supporting information on cell cycle distribution after 24 h treatment with different copper compounds in BEAS-2B cells. (PPTX 98 kb) [file 12989_2017_209_MOESM3_ESM.pptx]

## Slide 1
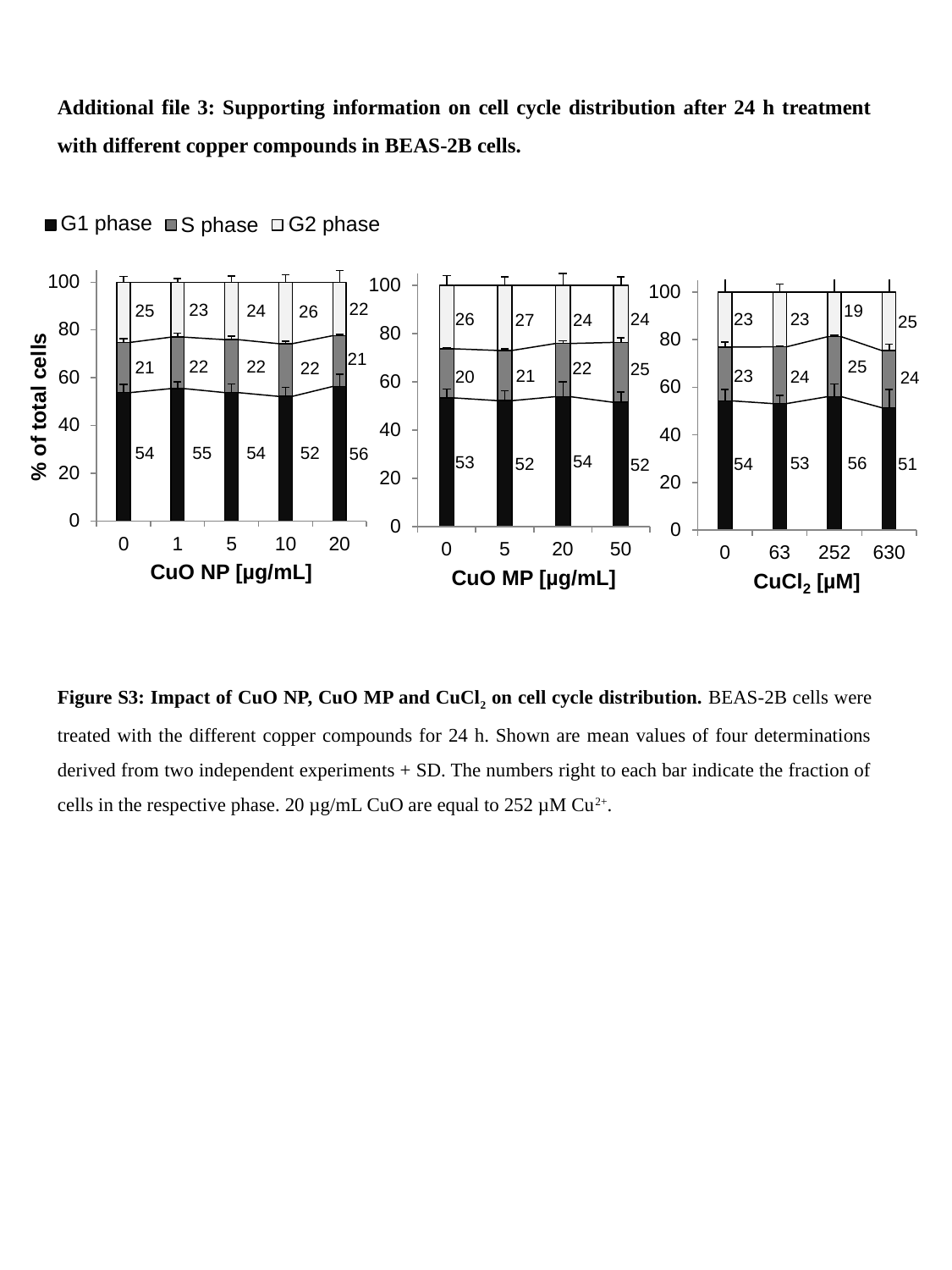

Additional file 3: Supporting information on cell cycle distribution after 24 h treatment with different copper compounds in BEAS-2B cells.
Figure S3: Impact of CuO NP, CuO MP and CuCl2 on cell cycle distribution. BEAS-2B cells were treated with the different copper compounds for 24 h. Shown are mean values of four determinations derived from two independent experiments + SD. The numbers right to each bar indicate the fraction of cells in the respective phase. 20 µg/mL CuO are equal to 252 µM Cu2+.
