# Supplementary material for: Comparison between micro- and nanosized copper oxide and water soluble copper chloride: interrelationship between intracellular copper concentrations, oxidative stress and DNA damage response in human lung cells
Source: Part Fibre Toxicol. 2017 Aug 1;14:28. doi: 10.1186/s12989-017-0209-1 (PMC5540434; doi:10.1186/s12989-017-0209-1)
Supplement: Supplementary file 4 — Supporting information on cell death induction after 24 h treatment with different copper compounds in BEAS-2B cells. (PPTX 81 kb) [file 12989_2017_209_MOESM4_ESM.pptx]

## Slide 1
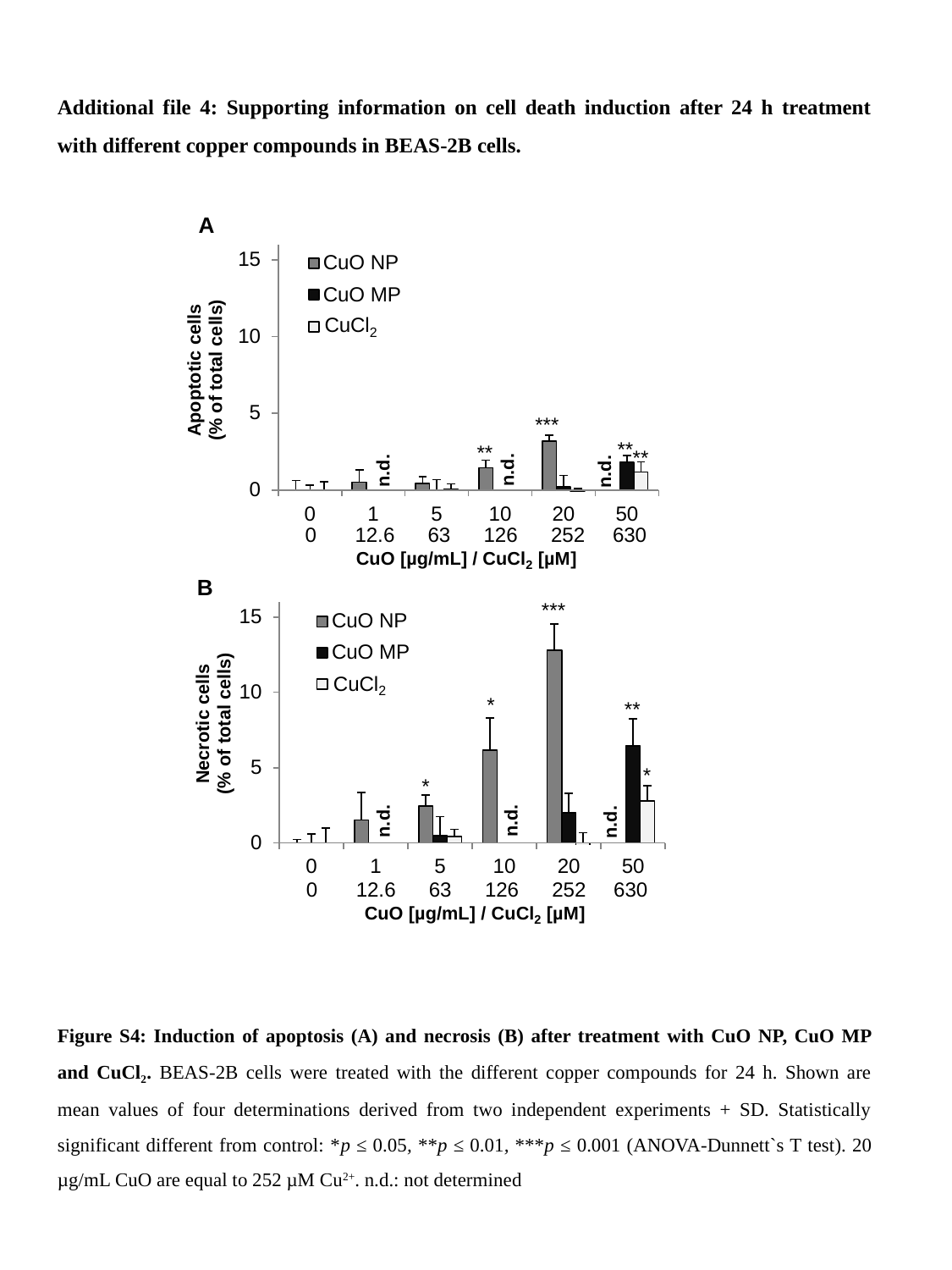

Additional file 4: Supporting information on cell death induction after 24 h treatment with different copper compounds in BEAS-2B cells.
Figure S4: Induction of apoptosis (A) and necrosis (B) after treatment with CuO NP, CuO MP and CuCl2. BEAS-2B cells were treated with the different copper compounds for 24 h. Shown are mean values of four determinations derived from two independent experiments + SD. Statistically significant different from control: *p ≤ 0.05, **p ≤ 0.01, ***p ≤ 0.001 (ANOVA-Dunnett`s T test). 20 µg/mL CuO are equal to 252 µM Cu2+. n.d.: not determined
